# A Cost of Illness Analysis of Children with Encephalitis Presenting to a Major Hospital in Vietnam

**DOI:** 10.4269/ajtmh.24-0409

**Published:** 2024-11-19

**Authors:** Nguyen Hoang Thien Huong, Nguyen Duc Toan, Truong Huu Khanh, Le Quoc Thinh, Le Nguyen Thanh Nhan, Ngo Ngoc Quang Minh, Nguyen Thi Kim Thoa, Nguyen Thanh Hung, Du Tuan Quy, C. Louise Thwaites, Sarosh R. Irani, Le Van Tan, Hugo C. Turner

**Affiliations:** ^1^ Emerging Infections Group, Oxford University Clinical Research Unit, Ho Chi Minh City, Vietnam;; ^2^ Clinical Departments, Children’s Hospital 1, Ho Chi Minh City, Vietnam;; ^3^ Department of Pediatrics, University of Health Sciences, Vietnam National University, Ho Chi Minh City, Vietnam;; ^4^ Department of Pediatrics, Pham Ngoc Thach University of Medicine, Ho Chi Minh City, Vietnam;; ^5^ Nuffield Department of Medicine, University of Oxford, Oxford, United Kingdom;; ^6^ Departments of Neurology and Neurosciences, Mayo Clinic, Jacksonville, Florida;; ^7^ Medical Research Council Centre for Global Infectious Disease Analysis, School of Public Health, Imperial College London, London, United Kingdom

## Abstract

Encephalitis is a significant global health problem, especially in children. Knowledge of its economic burden is essential for policymakers in prioritizing the development and implementation of interventions but remains limited. An observational study was prospectively conducted at a major children’s hospital in Ho Chi Minh City, Vietnam from 2020 to 2022. Data on direct medical costs, direct nonmedical costs, and productivity costs were collected alongside demographic information, clinical features, diagnosis, severity, and outcomes of study participants. This was used to undertake a cost of illness analysis from a societal perspective. Data were collected from a total of 164 pediatric patients. The median cost of illness was estimated at US $1,859 (interquartile range [IQR]: US $1,273–$3,128). The direct costs were the main cost driver, accounting for 83.9% of the total cost of illness (US $1,560; IQR: US $975–$2,460). The productivity costs accounted for a median of US $275 (IQR: US $154–$474). The cost of illness was higher in more severe patients, patients with sequelae, patients with morbidities, and ventilated patients. Most direct medical costs were attributed to hospitalization and resulted in out-of-pocket payments from the patient’s family (30.2%; US $316). The results showed that the cost of illness of encephalitis in children is considerable and will be useful for policymakers in prioritizing resources for the development and implementation of intervention strategies to reduce the burden of pediatric encephalitis.

## INTRODUCTION

Encephalitis is responsible for a significant global burden of disease, especially in children.^1^ An estimated 4–10 children per 100,000 are hospitalized with encephalitis every year,^2^^–^^4^ and it is associated with mortality rates of 2–31%.^5^^–^^7^ In a retrospective analysis of encephalitis cases in children living in England and Wales, the annual mortality rate was 0.07 (95% CI: 0.05–0.08) per 100,000 population of those between 0 and 17 years old.^8^ Among 7,298 children admitted with encephalitis between 2004 and 2013 in the United States, 2,933 patients (40%) required treatment in a pediatric intensive care unit and had an overall median length of hospital stay of 16 days.^4^ This high requirement for specialized treatment and lengthy hospital stay is linked to the high cost of encephalitis treatment.^2^^,^^4^^,^^9^^–^^11^

Data from the United States based on a nationally representative database of hospitalizations showed that the total cost of encephalitis-associated hospitalizations was estimated to be US $2.0 billion in 2010^12^ and that the average economic burden of a child with encephalitis was estimated to be between US $64,000 and $260,000 depending on the level of health care and rehabilitation.^2^^,^^4^^,^^10^ In low- and middle-income countries where encephalitis in children is common, there are only limited data available.^13^^–^^15^ A longitudinal follow-up study of acute encephalitis syndrome in children from two hospitals in Nepal^13^ found that the median out-of-pocket cost to families, including medical bills, medication, and lost earnings, was US $1,151 for children with severe/moderate impairment and US $524 for those with mild/no impairment.^13^ In a Cambodian study that interviewed affected families, it was found that costs related to acute hospital admission were the major cost driver.^14^ Medication was identified to be the predominant acute medical cost associated with pediatric encephalitis in Indonesia.^15^

A previous cost of illness analysis of Japanese encephalitis in Vietnam estimated a mean total cost of US $3,371 per acute episode.^16^ This showed that patients with Japanese encephalitis and their families in Vietnam suffer notable medical, economic, and social hardship.^16^ Although this study has provided valuable insight into the costs of one cause of encephalitis in Vietnamese children, knowledge regarding the economic burden of pediatric encephalitis more generally is lacking, but it is essential to inform local policymakers in planning and prioritizing resources for diagnosis and treatment and public health interventions. For this reason, we carried out this study, conducting a prospective observational study to estimate the costs attributed to pediatric encephalitis in a major hospital in southern Vietnam.

## MATERIALS AND METHODS

### Study design and setting.

The study was a prospective observational study conducted at Children’s Hospital 1 (CH1) in Ho Chi Minh City in Vietnam between March 2020 and December 2022.^17^ CH1 is a 1,600-bed hospital, and it is the largest tertiary hospital for children living in the southern provinces of Vietnam, with a catchment population of over 40 million. Annually, CH1 has approximately 90,000 admissions. Of these, approximately 150–200 cases receive a diagnosis of encephalitis.

### Inclusion and exclusion criteria.

Any child admitted to the Department of Infectious Diseases and Neurology of CH1 and fulfilling the case definition of encephalitis between March 2020 and December 2022^17^^–^^19^ was eligible for study participation. Patients were excluded if no informed consent was obtained. Given the descriptive nature of the present study, we used a convenience sampling approach, and no formal sample size calculation was performed.

### Case definition of encephalitis.

In our study, encephalitis in children^17^^–^^19^ was diagnosed when the patient had altered mental status (i.e., decreased or altered level of consciousness, lethargy, or personality change) lasting 24 hours or longer with no alternative cause identified and two or more of the following criteria, including documented fever ≥38°C (100.4°F) within 72 hours of presentation (before or after), generalized or partial seizures not fully attributable to a preexisting seizure disorder, new-onset focal neurologic findings, cerebrospinal fluid (CSF) white blood cell count ≥5 cells/mm,^3^ abnormality of brain parenchyma on neuroimaging suggestive of encephalitis that was new or appeared to have acute onset, and abnormality on electroencephalogram that was consistent with encephalitis and not attributable to any other causes.^17^^–^^19^

### Data collection.

Clinical data, including dates of birth, admission and discharge, demographic data, clinical features, routine diagnosis, and outcomes, were collected from all participants. The definitions of clinical outcomes in our study can be found in Supplemental Table 1. Patient consciousness level was evaluated using the pediatric Glasgow coma scale (GCS), and those with GCS less than nine were categorized as having severe disease.^20^ The modified Rankin scale (mRS) for children was used to assess the outcomes in terms of degree of disability or dependence in daily activities.^21^^–^^23^ The mRS for children is currently used to measure the hospital outcomes of patients in the practice at our institution and has been used in other similar populations internationally.^22^^,^^23^ The mRS scores have been dichotomized for practical use, but currently, there is no consistent method of dichotomization as there is no consensus regarding good or poor outcome using mRS.^24^ In our study, mRS scores greater than or equal to three were categorized as “more severe” disability, whereas mRS scores less than three were categorized as “less severe” disability; this is similar to the current approach in many studies.^25^^–^^28^ The neurological sequelae and disability were assessed immediately on hospital discharge by two qualified neurologists. If there was disagreement, then the diagnosis of neurological sequelae was discussed until a consensus decision was reached. Neurological sequelae because of encephalitis included paralysis, developmental delay, altered mental status, speech disorder, and motor disturbance. Requiring respiratory support with mechanical ventilation was used as a proxy indicator of disease severity.^9^^,^^29^^–^^31^ In our study, acute extraneurological symptoms/signs were defined as acute extraneurological or noncentral nervous system symptoms/signs, and these were not preexisting diseases. Data were collected from hospital invoices and structured questionnaires administered through face-to-face interviews. The hospital invoices show details of the cost paid by insurance and by the patients for all cases. Caregivers were interviewed at admission (for the costs before hospital admission), just before hospital discharge (for the costs during hospitalization), and at the outpatient clinic of the hospital (for the costs up to 7 days after discharge). We did not carry out telephone interviews to follow up on these patients.

### Laboratory diagnosis.

As part of routine care at CH1, encephalitis diagnosis was made through analysis of CSF, including culture and/or microscopy for detection of bacterial infections. Patients with suspected encephalitis were further tested for herpes simplex virus and Japanese encephalitis virus using polymerase chain reaction and serology, respectively. Patients with clinical presentations compatible with autoimmune encephalitis were tested for possible forms of the disease using commercial fixed cells-based assays (EUROIMMUN, Lübeck, Germany).^32^ Autoantibody testing was conducted on the CSF samples only.

### Cost evaluation.

The total cost of illness consisted of direct medical costs, direct nonmedical costs, and productivity costs. The costs were collected from a societal perspective. Data on the cost of illness were collected by capturing the periods before hospital admission, during hospitalization, and 7 days after discharge.

The direct medical costs consist of the costs directly associated with the use of medical resources, goods, and services (such as the costs associated with diagnostic tests, drugs, etc.). These costs were calculated based on the hospital invoices and the information obtained from the face-to-face interviews. The direct medical costs attributed to hospitalization were collected based on the official invoices within the patients’ in-hospital medical records. We had official invoices for all prescribed medical items during hospitalization. These costs could be stratified by the cost covered by the government’s health insurance program and the family’s out-of-pocket payments. The direct medical costs incurred before hospital admission and up to 7 days after discharge were captured through interviews. However, because these were not based on official invoices, it was not possible to stratify these costs depending on whether they were paid by the families themselves or covered by the health insurance program.

The direct nonmedical costs represent the costs related to the use of nonmedical resources. These include the patients’/caregivers’ travel costs and other expenses related to the care of the patient. Data on direct nonmedical costs were collected through face-to-face interviews.

The productivity costs represent the value of monetized productivity losses resulting from lost paid and unpaid work because of an illness or an intervention. Within this study, only the productivity losses of the caregivers were considered and valued. The productivity losses of children (such as from missed school days) were not valued. Data on the caregivers’ productivity losses incurred were collected through face-to-face interviews by asking about the number of days that they lost because of caregiving before hospital admission, during hospitalization, and up to 7 days after discharge. How these productivity losses were valued depended on the activity that the informal caregivers reported giving up. For those who reported giving up paid employment, their losses were valued based on their reported monthly salary. For those who reported losing unpaid work, their losses were valued based on the minimum wage. As the minimum wage in Vietnam varies across the different provinces (Supplemental Table 2),^33^ we used the monthly minimum wage that corresponded to the address (province) of the patient. The monthly salary and minimum wage values were adjusted to a daily value based on the caregivers’ reported number of working days per month (Supplemental Table 3). The productivity costs were calculated based on this daily value multiplied by the reported number of days lost.

Because of the local coronavirus disease 2019 (COVID-19) regulations during the study, only one registered caregiver (usually the father or the mother) was allowed to stay with and take care of the child, and the caregiver did not change during the hospitalization period. Therefore, the productivity costs were estimated only for one registered caregiver of a patient. No excess in-hospital death was considered in the calculation of productivity costs.

Under the government health insurance scheme in Vietnam, children under 6 years old are provided with free health care services. For children 6–14 years old, there are two main health insurance programs, which are operated by the Vietnam Health Insurance Organization on a nonprofit and public basis. However, the families have to pay for medical services that are not listed in the health insurance directory issued by the Ministry of Health.^34^

Our cost data were highly skewed by using skewness and a kurtosis test for normality. Comparisons between groups were made using the statistical nonparametric test, which was the Wilcoxon rank-sum test. We summarized all values of illness costs as median and interquartile range (IQR) in US dollars, with a conversion rate of US $1 equivalent to 23,271.2 dong (the exchange rate between Vietnamese dong and US dollars for the year 2022) based on the database from the World Bank. Costs incurred in 2020 and 2021 were adjusted to 2022 prices using gross domestic product deflators for Vietnam using the approach outlined within the work of Turner et al.^35^

## RESULTS

### Baseline features of children with encephalitis.

**Figure 1.**
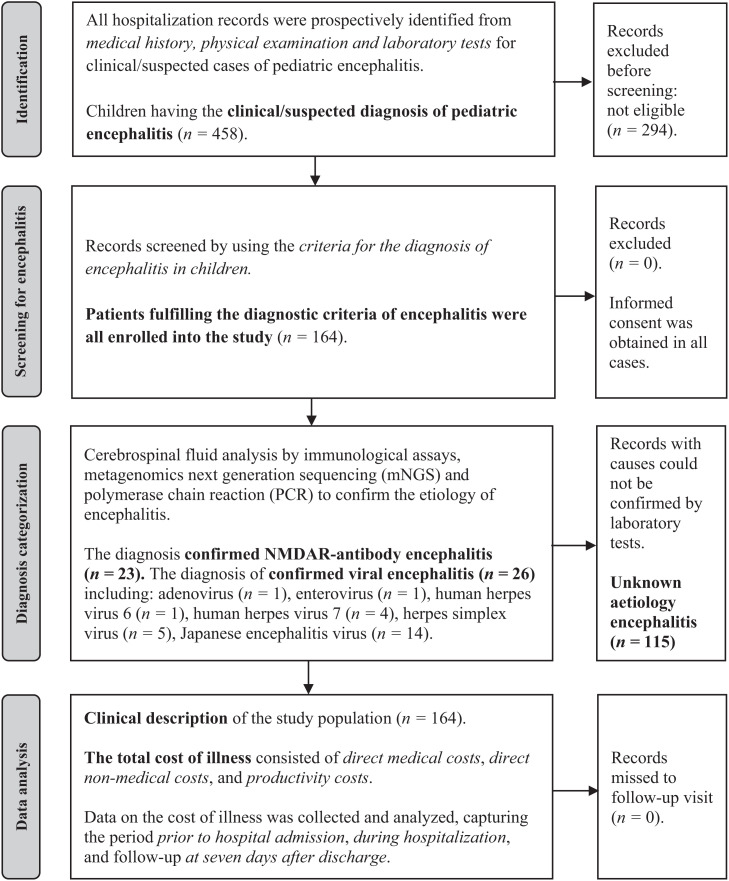
Screening, enrollment, and follow-up of patients with encephalitis. NMDAR = N-methyl-D-aspartate receptor.

Between March 2020 and December 2022, 164 children with clinical features of encephalitis were enrolled in the study. Among these, 23 of 164 patients (14.0%) were confirmed as having N-methyl-D-aspartate receptor (NMDAR)-antibody encephalitis. Viral encephalitis was diagnosed in 26 of 164 cases (15.9%). Encephalitis caused by Japanese encephalitis virus (JEV) was identified in 14 of 164 cases (8.5%). The key clinical features of NMDAR-antibody encephalitis and JEV are shown in Supplemental Table 4. The other cases were reported as unknown infectious etiology encephalitis (115/164; 70.1%) as the etiologies of these cases could not be identified (Table 1). The baseline features of children with encephalitis are shown in Table 1. All patients in our study did not miss follow-up visits, and all had data at 7 days after discharge. The screening, enrollment, and follow-up of patients are showed in Figure 1.

**Table 1:** Clinical features of the patients

Features of Patients with Encephalitis	NMDAR-Antibody Encephalitis (*N* = 23)	Confirmed Viral Encephalitis (*N* = 26)	Unknown Etiology Encephalitis (*N* = 115)
General features			
Sex			
Male*	4 (17.4)	18 (69.2)	63 (54.8)
Female*	19 (82.6)	8 (30.8)	52 (45.2)
Age (years)^†^	10 (9–13)	8 (3–12)	9 (6–12)
Residence			
Ho Chi Minh City*	7 (30.4)	2 (7.7)	34 (29.6)
Other provinces*	16 (69.6)	24 (92.3)	81 (70.4)
Clinical features			
GCS score^†^	11 (9–12)	9 (7–11)	12 (10–13)
GCS <9* (more severe)	4 (17.4)	13 (50.0)	9 (7.8)
GCS ≥9* (less severe)	19 (82.6)	13 (50.0)	106 (92.2)
Acute extraneurological symptoms/signs*	18 (78.3)	15 (57.7)	63 (54.8)
Outcomes			
Respiratory support with ventilation*	6 (26.1)	13 (50.0)	12 (10.4)
mRS score^†^	2 (0–3)	1 (0–2)	0 (0–1)
mRS <3* (less severe)	16 (69.6)	23 (88.5)	102 (88.7)
mRS ≥3* (more severe)	7 (30.4)	3 (11.5)	13 (11.3)
Sequelae*	13 (56.5)	16 (61.5)	35 (30.4)
In-hospital death*	1 (4.3)	0 (0.0)	2 (1.7)
Duration of hospital stay (days)^†^	38 (15–53)	12 (10–22)	15 (10–22)

GCS = Glasgow coma scale; mRS = modified Rankin scale.

* Two groups of GCS and mRS score outputs are presented as *N* (percentage).

^†^ The GCS and mRS score outputs are presented as median (interquartile range).

### The informal caregivers’ characteristics.

The most common normal activities that the caregivers reported giving up to provide care for the child were paid employment (76/164; 46.3%), housework (36/164; 22.0%), and subsistence farming (32/164; 19.5%). The median number of days lost was 28 days (IQR: 20–43 days). The productivity losses were stratified depending on whether they were incurred before hospitalization, during hospitalization, or after hospitalization. The median monthly income of the caregivers was US $201 (IQR: US $156–$430) (Table 2).

**Table 2:** Information regarding the caregivers

Characteristics (*N* = 164*)	*N* (%) or Median (IQR)
Residence, *N* (%)	
Ho Chi Minh City	43 (26.2)
Other provinces	121 (73.8)
Relationship, *N* (%)	
Mother	118 (72.0)
Father	46 (28.0)
Normal activities, *N* (%)	
Paid employment	77 (46.9)
Business ownership	19 (11.6)
Subsistence farming (unpaid work)	32 (19.5)
Housework (unpaid work)	36 (22.0)
Working days per month, median (IQR)	26 (24–26)
Working days per month of paid workers	26 (24–26)
Working days per month of unpaid workers	26 (22–28)
Days of work lost, median (IQR)	28 (20–43)
Days lost before hospitalization	4 (3–7)
Days lost during hospitalization	14 (9–23)
Days lost after hospitalization	7 (7–7)
Individual income per month (US dollars), median (IQR)	201 (156–430)

IQR = interquartile range.

* Only one registered caregiver could stay with the child during the hospitalization period.

### An overview of the costs of illness attributed to pediatric encephalitis.

A summary of the estimated costs associated with encephalitis in children is presented in Table 3. The total median cost of illness was US $1,859 (IQR: US $1,273–$3,128). The direct costs constituted the majority (83.9%) of this total cost of illness. The median was US $1,560 (IQR: US $975–$2,460), of which the direct medical cost incurred during hospitalization was found to be the main component/driver, with a median of US $1,044 (IQR: US $615–$1,906). Importantly, in Vietnam, the government’s health insurance program does not cover all of the medical costs, and in this study, the patients had to pay 30.2% (US $316) of the direct medical costs incurred during hospitalization. The median direct nonmedical costs were US $335 (IQR: US $226–$505), 37.0% of which were related to transportation. The remaining accounted for other types of direct nonmedical costs, such as costs related to the care of the patient. The productivity costs during hospitalization were the dominant contributor to the total productivity costs (US $138 of $275).

**Table 3:** Summary of the estimated costs of illness of pediatric encephalitis cases

Costs Type	Median US Dollars (IQR)
Direct medical cost	1,202 (698–2,087)
Direct medical costs before hospital admission	34 (0–134)
Direct medical costs during hospitalization	1,044 (615–1,906)
Direct medical costs up to 7 days after discharge	43 (21–46)
Direct medical cost paid by the patients	
Direct medical costs before hospitalization paid by the patients	NA
Direct medical costs during hospitalization paid by the patients	316 (154–623)
Direct medical costs during hospitalization paid by the patients younger than 6 years of age (*n* = 41)	223 (86–645)
Direct medical costs during hospitalization paid by the patients 6 years of age or older (*n* = 123)	326 (183–602)
Direct medical costs up to 7 days after discharge paid by the patients	NA
Direct medical cost paid by health insurance	
Direct medical costs before hospitalization paid by health insurance	NA
Direct medical costs during hospitalization paid by health insurance	631 (330–1,117)
Direct medical costs during hospitalization paid by health insurance for patients younger than 6 years of age (*n* = 41)	873 (488–1,364)
Direct medical costs during hospitalization paid by health insurance for patients 6 years of age or older (*n* = 123)	570 (261–1,034)
Direct medical costs up to 7 days after discharge paid by health insurance	NA
Direct nonmedical cost	335 (226–505)
Transportation costs before hospital admission	9 (0–43)
Transportation costs during hospitalization	92 (45–138)
Transportation costs up to 7 days after discharge	23 (9–46)
Other expenses*	172 (92–275)
Total productivity cost	275 (154–474)
Productivity costs before hospital admission	42 (23–89)
Productivity costs during hospitalization	138 (77–280)
Productivity costs up to 7 days after discharge	68 (42–116)
Total direct cost	1,560 (975–2,460)
Total costs of illness (societal perspective)	1,859 (1,273–3,128)

IQR = interquartile range; NA = not available.

* Other expenses are other costs related to the care of the patient.

### The valuation of productivity costs.

Paid work constituted 58.5% (96/164) of the normal activities of the caregivers. The median productivity costs for caregivers who lost paid work were estimated to be US $368 based on their reported wages. For those who lost unpaid work, the median productivity costs were calculated as US $188 based on the daily minimum wage. Consequently, the productivity costs of paid workers are estimated to be twice as much as caregivers performing unpaid work (Table 4). The productivity loss during hospitalization contributed to approximately half of the total productivity loss of caregivers (Table 4).

**Table 4:** The valuation of the caregivers’ productivity costs

Productivity Costs and Related Factors	Caregivers Losing Paid Work,* Median (IQR)	Caregivers Losing Unpaid Work,^†^ Median (IQR)
Monthly income (US dollars)	344 (236–430)	156 (156–201)
Working days per month	26 (24–26)	26 (22–28)
Daily value of a lost day^‡^ (US dollars)	15 (10–17)	7 (6–8)
Days lost before hospitalization	5 (3–7)	4 (3–7)
Days lost during hospitalization	15 (9–23)	12 (8–24)
Days lost after hospitalization	7 (7–7)	7 (7–7)
Total days lost	29 (20–43)	26 (19–43)
Productivity cost before hospitalization (US dollars)	53 (33–105)	28 (19–51)
Productivity cost during hospitalization (US dollars)	185 (114–329)	90 (53–157)
Productivity cost after hospitalization (US dollars)	103 (58–125)	49 (39–68)
Total productivity cost (US dollars)	368 (229–642)	188 (122–305)

IQR = interquartile range.

* For those who reported losing paid work, their productivity losses were valued based on the monthly salary (*N* = 96).

^†^ For those who reported losing unpaid work, their productivity losses were valued based on the monthly minimum wage (*N* = 68).

^‡^ This value was calculated by dividing the monthly income (salary for lost paid work and minimum wage for lost unpaid work) by the reported number of working days per month (Supplemental Table 2). The productivity cost was calculated by multiplying this daily value by the number of days lost.

### Costs of illness of encephalitis in children by geographic location, diagnosis, severity, and outcomes.

Ventilated patients had higher total costs of illness than nonventilated patients (median, US $3,162 versus median, US $1,754; *P* <0.0001). Assessing the costs by the degree of disability or dependence in their daily activities, the total costs of illness at discharge of more severely affected patients (mRS greater than or equal to three) were much higher than those of less severely affected patients (mRS less than three; median, US $6,193 compared with US $1,763; *P* <0.0001). Similarly, patients with sequelae had higher total costs compared with those of patients without sequelae (median, US $2,359 versus median, US $1,721; *P* = 0.0007) (Table 5). We identified a number of factors that influenced the projected costs of illness (Table 5). Patients with confirmed NMDAR-antibody encephalitis were associated with higher costs of illness (median, US $2,823) compared with other categories of encephalitis in children (*P* = 0.0003). As expected, in terms of the level of consciousness, the total costs of illness of more severe patients were higher than those of less severe patients (median, US $2,685 versus median, US $1,775; *P* = 0.0006). Patients with acute extraneurological symptoms/signs had higher costs of illness compared with those who did not have acute extraneurological symptoms/signs (median, US $2,497 versus median, US $1,344; *P* <0.0001).

**Table 5:** Estimated costs of illness stratified by different patient groupings

Patient Groups*	Direct Medical Costs, Median US Dollars (IQR)	Direct Nonmedical Costs, Median US Dollars (IQR)	Total Direct Costs, Median US Dollars (IQR)	Total Productivity Costs, Median US Dollars (IQR)	Total Costs of Illness, Median US Dollars (IQR)	*P*-Value^†^
All patients (*N* = 164)	1,211 (698–2,087)	335 (226–505)	1,560 (975–2,460)	275 (154–474)	1,859 (1,273–3,128)	ND
Age of patients						
<6 years of age (*n* = 41)	1,364 (780–2,217)	460 (290–602)	1,885 (1,172–2,558)	259 (151–532)	2,018 (1,466–3,144)	0.2081
≥6 years of age (*n* = 123)	1,160 (692–1,964)	312 (206–438)	1,514 (959–2,425)	284 (161–434)	1,837 (1,169–3,041)	
Residence of patients						
Ho Chi Minh City (*n* = 43)	1,205 (652–2,551)	258 (215–390)	1,460 (882–2,941)	298 (116–469)	1,754 (1,141–3,336)	0.6309
Other provinces (*n* = 121)	1,199 (718–1,964)	374 (250–536)	1,643 (1,034–2,425)	275 (163–481)	1,935 (1,309–3,041)	
Diagnosis of pediatric encephalitis						
NMDAR-antibody encephalitis (*n* = 23)	1,760 (1,332–5,746)	580 (298–735)	2,133 (1,830–6,365)	550 (299–925)	2,823 (2,225–7,573)	**0.0003** ^‡^
Confirmed viral encephalitis (*n* = 26)	1,363 (780–2,116)	294 (223–394)	1,693 (1,055–2,479)	196 (130–360)	1,998 (1,181–2,759)	0.9175^§^
Unknown etiology encephalitis (*n* = 115)	1,106 (661–1,705)	326 (220–455)	1,460 (954–2,029)	266 (157–434)	1,763 (1,201–2,563)	**0.0073** ^ǁ^
Acute extraneurological symptoms/signs						
With acute extraneurological symptoms/signs (*n* = 96)	1,569 (1,085–3,947)	392 (296–597)	1,960 (1,370–4,562)	336 (190–673)	2,497 (1,708–5,288)	**<0.0001**
Without acute extraneurological symptoms/signs (*n* = 68)	771 (572–1,204)	248 (182–363)	1,051 (779–1,547)	242 (134–369)	1,344 (987–1,889)	
GCS						
GCS <9 (more severe; *n* = 26)	2,036 (1,338–6,009)	414 (289–589)	2,409 (1,676–6,634)	321 (161–481)	2,685 (1,839–7,052)	**0.0006**
GCS ≥9 (less severe; *n* = 138)	1,115 (660–1,705)	323 (220–481)	1,475 (954–2,133)	275 (151–469)	1,775 (1,181–2,700)	
mRS						
mRS <3 (less severe; *n* = 141)	1,128 (660–1.684)	309 (223–430)	1,496 (954–2,029)	247 (144–395)	1,763 (1,181–2,437)	**<0.0001**
mRS ≥3 (more severe; *n* = 23)	4,227 (1,332–7,267)	619 (509–784)	4,846 (1,983–8,135)	734 (367–1,284)	6,193 (3,144–8,255)	
Respiratory support with ventilators						
Ventilated (*n* = 31)	2,408 (1,338–6,009)	430 (289–625)	2,752 (1,676–6,634)	323 (161–627)	3,162 (1,839–7,052)	**<0.0001**
Nonventilated (*n* = 133)	1,084 (659–1,621)	312 (220–460)	1,460 (948–2,029)	275 (151–465)	1,754 (1,169–2,563)	
In-hospital death						
Died (*n* = 3)	1,205 (721–6,009)	193 (142–688)	1,398 (863–6,697)	297 (81–319)	1,696 (944–7,016)	0.8492
Survived (*n* = 161)	1,199 (692–2,057)	344 (236–501)	1,564 (976–2,440)	275 (157–479)	1,861 (1,284–3,111)	
Sequelae						
With sequelae (*n* = 64)	1,393 (1,036–3,846)	379 (264–595)	1,883 (1,350–4,479)	377 (219–719)	2,359 (1,664–5,288)	**0.0007**
Without sequelae (*n* = 100)	1,072 (652–1,784)	317 (211–455)	1,348 (904–2,165)	236 (139–380)	1,721 (1,143–2,397)	

GCS = Glasgow coma scale; IQR = interquartile range; mRS = modified Rankin scale; ND = not done; NMDAR = N-methyl-D-aspartate receptor.

* Data are presented as median (IQR).

^†^ Comparisons of total costs of illness between groups are made using the Wilcoxon rank-sum test.* P* <0.05 is considered to be statistically significant, and these values are highlighted in bold.

^‡^ Comparisons of total costs of illness between NMDAR-antibody encephalitis and other categories of encephalitis.

^§^ Comparisons of total costs of illness between confirmed viral encephalitis and other categories of encephalitis.

^ǁ^ Comparisons of total costs of illness between unknown etiology encephalitis and other categories of encephalitis.

## DISCUSSION

Despite the clear presence and clinical threat of pediatric encephalitis in Vietnam, limited information regarding its economic burden is available to support policymakers and physicians in prioritizing the resources for the improvement and execution of intervention strategies. Here, we describe the results of a prospective hospital-based study during 2020–2022 that estimated the cost of illness of encephalitis in children. Among 458 patients with clinical/suspected cases of pediatric encephalitis, only 164 cases (35.8%) fulfilled the diagnostic criteria of encephalitis; these patients were all enrolled in the study. This may reflect the significant contribution of encephalitis (35.8%) to the diagnosis profile of patients with initial signs and symptoms of central nervous system infections at our center during the study period or the stricter definition used for our study compared with hospital diagnostic coding.

The results show that pediatric encephalitis cases are associated with a substantial economic burden in Vietnam. Direct costs were the main cost driver, accounting for 84.6% of the total cost, particularly the direct medical cost during hospitalization. In terms of the direct nonmedical costs, the transportation costs during hospitalization and expenses related to childcare arrangements were the main drivers. Patients with NMDAR-antibody encephalitis, more severely ill patients, and patients with sequelae had higher total costs. This may be because of the longer length of stay needed for these patients (Table 1). Our analysis has found that cases with acute extraneurological symptoms/signs had higher costs than those without acute extraneurological symptoms/signs. However, we believe that this reflects multisystemic disease (i.e., more severe disease) and would be expected to be associated with higher costs.

Importantly, the total median direct medical costs associated with nonventilated and ventilated children suffering from encephalitis in our study were approximately 6.5 times and 14.5 times higher, respectively, than Vietnam’s annual average per capita health care spending in 2020 (US $166.2, 2020 prices). The direct medical costs during hospitalization paid by the patients themselves (not covered by government health insurance) were approximately 1.6 times higher than the income per month. We also found that families incur notable direct nonmedical costs (with a median of US $335). These direct nonmedical costs are not covered by insurance, and therefore, the families have to pay for these expenses themselves. These numbers are concerning and highlight the risk of families incurring catastrophic health expenditures. In addition, the productivity cost during the hospitalization period was also much higher than the minimum earnings of the parent, posing an important loss to the family and society.

Our study only looked at the acute phase of encephalitis. However, persistent neurologic effects are common after encephalitis: for example, personality change, behavioral disorders, movement disorders, intellectual disability, learning disorders, blindness, paresis, and sleeping problems.^36^^,^^37^ Such disorders are likely to have long-lasting economic impacts to individuals, families, and society. In addition, our study has other limitations. First, it only investigated patients admitted to one hospital in Ho Chi Minh City, Vietnam. These cost estimates cannot necessarily be generalized to every encephalitis case in Vietnam. For example, as a specialist center, it is possible that the severity of cases and association with long-term sequelae may be higher in our sample. It is important to note that the current/standard approach to monetize productivity losses remains an area of debate, particularly regarding the valuation of unpaid work and whether to include the valuation of the productivity losses of children.^38^^–^^40^ In addition, it is possible that some cost items could have been missed (such as any direct medical costs that were not on the hospital invoices). The local COVID-19 regulations during the study period likely reduced the number of caregivers per patient and the associated costs associated with the caregivers. During the COVID-19 pandemic, the number of patients with encephalitis admitted to our hospital was reduced. Therefore, the COVID-19 pandemic may have affected the representativeness of the patients enrolling into our study (e.g., fewer referrals from rural areas because of the lockdown, which may have had an impact on the epidemiological findings and the generalizability of the results of this study). Finally, the cost data estimates calculated within this paper were based directly on the charges from the patients’ hospital bills, and the costs related to the staff time were assumed to be captured by the charges for the different services. However, these charges do not necessarily reflect the economic value of the resources used for their care.^41^^–^^43^ To try and capture economic costs within this context, a cost-to-charge ratio is commonly applied to the charges (which is based on the ratio of the hospital’s [or department’s] expenses and what they charge),^44^^,^^45^ but the data were not available to do this adjustment within this study.

## CONCLUSION

Our results show that the cost of illness of encephalitis in children is considerable and higher in more severe patients, patients with sequelae, and ventilated patients. Notably, we found that despite high health insurance coverage, patients and families still incur significant costs. Of note, many of the children in our study suffered from JEV (14/164; 8.5%), a vaccine-preventable disease, indicating the potential of preventative public health measures to impact and reduce these cost outcomes.

## Supplemental Materials

10.4269/ajtmh.24-0409 Supplemental Materials
