# Supplementary material for: A Cost of Illness Analysis of Children with Encephalitis Presenting to a Major Hospital in Vietnam
Source: Am J Trop Med Hyg. 2024 Nov 19;112(2):422–30. doi: 10.4269/ajtmh.24-0409 (PMC11803661; doi:10.4269/ajtmh.24-0409)
Supplement: Supplemental Materials [file tpmd240409.SD1.pdf]

Supplementary Table S1. Pediatric Glasgow coma scale and modified Rankin scale

	**Definitions**	**Definitions of more severe outcomes in our study**
**The pediatric Glasgow coma scale (GCS)**	The pediatric Glasgow coma scale (GCS) was used in this study to assess the impaired consciousness and coma in paediatric patients with encephalitis. The GCS comprises three examinations including eye, verbal and motor responses. The three values separately as well as their sum are considered. The lowest possible sum of GCS is 3 (deep coma or death) whilst the highest is 15 (fully awake and aware person).	We used the Paediatric Glasgow coma scale (GCS) to evaluate the consciousness, and the patients were defined to have more severe condition when the GCS < 9.
**The modified Rankin scale (mRS) for children**	The modified Rankin scale (mRS) for children was used in this study to measure the degree of disability or dependence in the daily activities of children who have suffered encephalitis. The scale comprises seven levels, from 0 to 6, with higher scores indicating greater disability and where 0–2 is generally considered a good outcome with individuals assuming complete functional independence. A modified Rankin score of 6 is often used to denote an individual who is deceased.	We used modified Rankin scale (mRS) for children to assess the degree of disability or dependence in the daily activities, and the patients were defined to have more severe condition when the mRS ≥ 3.

Supplementary Table S2. Monthly minimum wages in four regions in Vietnam

Region	Provinces	Minimum wages per month (US$)
**I**	Ho Chi Minh, Binh Duong, Dong Nai, Vung Tau, Can Tho	201
**II**	Hue, Tay Ninh	179
**III**	Binh Thuan, Binh Phuoc, Long An, Tien Giang, An Giang, Kien Giang, Ca Mau, Ben Tre, Bac Lieu, Lam Dong, Khanh Hoa, Hau Giang, Vinh Long, Quang Ngai	156
**IV**	Tra Vinh, Soc Trang, Dak Nong, Dak Lak, Dong Thap	140

Supplementary Table S3. Valuation of productivity losses

Scenarios[*]	Monthly income (US$)	Reported working days per month (days)	Daily value of a lost day used to monetize the productivity losses (US$)
**Scenario 1** (n = 96): For caregivers that reported losing paid employment, productivity losses were valued based on their reported monthly salary. The daily value of a lost day was calculated based dividing the monthly salary by the reported number of working days per month	344 (236–430)	26 (24–26)	14 (10–17)
**Scenario 2** (n = 68): For caregivers that reported losing unpaid work, productivity losses were valued based on the minimum wage.<br>The daily value of a lost day was calculated based dividing the minimum wage by the reported number of working days per month	156 (156–201)	26 (22–28)	7 (6–8)

Abbreviation: IQR, interquartile range.
[*]All data are presented as median (IQR)

Supplementary Table S4. Clinical features of NMDAR-antibody encephalitis and JEV encephalitis

	**NMDAR-antibody encephalitis (n = 23)**	**JEV encephalitis (n = 14)**	***P*-values***
**Demographic features**			
Female	19 (82.6)	2 (14.3)	<0.001
Age (years)	10.0 (9.0–13.0)	4.5 (1.0–9.0)	0.007
Ho Chi Minh city	7 (30.4)	0 (0.0)	0.031
Other provinces	16 (69.6)	14 (100.0)	
**Clinical features**			
Illness days before admission	4 (3 – 10)	5 (3 – 6)	0.975
Fever	13 (56.5)	14 (100.0)	0.006
Highest temperature during hospitalization (^{o}C)	38.8 (38.5–39.0)	39.0 (39.0–40.0)	0.007
Duration of fever (days)	3 (3–7)	7 (5–10)	0.014
Glasgow coma scale (GCS)	11 (9–12)	8 (8–11)	0.036
Seizure	17 (73.9)	6 (42.9)	0.059
Neurologic deficits	6 (26.1)	7 (50.0)	0.139
Abnormal muscular tone	8 (34.8)	4 (28.6)	0.695
**MRI findings**			
Cerebral cortex	13 (56.5)	2 (14.3)	0.011
Limbic system	15 (65.2)	2 (14.3)	0.003
Thalamus	9 (39.1)	14 (100.0)	<0.001
Mid-brain	9 (39.1)	3 (21.4)	0.265
Cerebellum	7 (30.4)	3 (21.4)	0.550
Brain stem	4 (17.4)	1 (7.1)	0.377
**EEG abnormality**			
Delta brush	3 (13.0)	0 (0.0)	0.275
Slow waves	12 (52.2)	0 (0.0)	0.001
Spike waves	2 (8.7)	0 (0.0)	0.517
Beta waves	1 (4.3)	0 (0.0)	1.000
Seizure	1 (4.3)	0 (0.0)	1.000
Fast activity	2 (8.7)	0 (0.0)	0.517
Slow baseline activity	1 (4.3)	0 (0.0)	1.000
Low voltage	1 (4.3)	0 (0.0)	1.000
**Treatment**			
Corticosteroid	21 (91.3)	1 (7.1)	<0.001
Immunoglobulin	9 (39.1)	0 (0.0)	0.007
Cyclophosphamide	7 (30.4)	0 (0.0)	0.031
Rituximab	4 (17.4)	0 (0.0)	0.276
Acyclovir	18 (78.3)	10 (71.4)	0.639
Mannitol	14 (60.9)	12 (85.7)	0.109
Sodium chloride 3%	3 (13.0)	3 (21.4)	0.502
**Outcomes**			
Length of hospital stay (days)	38 (15–53)	15 (11–20)	0.021
Mortality	1 (4.3)	0 (0.0)	1.000
Sequelae	13 (56.5)	9 (64.3)	0.641
Modified Rankin scale (mRS)	2 (0–3)	1 (0–2)	0.553

Data are presented as n (%) or median (IQR).
*Comparisons are made between NMDAR-antibody encephalitis and JEV encephalitis using Pearson's chi-squared test, Fisher's exact test, Wilcoxon rank-sum test. A p-value less than 0.05 is statistically significant.
